# Genome-Wide Association Analysis for Salt–Induced Phenotypic and Physiologic Responses in Rice at Seedling and Reproductive Stages

**DOI:** 10.3389/fpls.2022.822618

**Published:** 2022-02-09

**Authors:** Gang Chen, Keming Hu, Jianhua Zhao, Feifei Guo, Wenfeng Shan, Qiuqing Jiang, Jinqiao Zhang, Zilong Guo, Zhiming Feng, Zongxiang Chen, Xiaoxia Wu, Shengwei Zhang, Shimin Zuo

**Affiliations:** ^1^Key Laboratory of Plant Functional Genomics of the Ministry of Education/Jiangsu Key Laboratory of Crop Genomics and Molecular Breeding, Agricultural College of Yangzhou University, Yangzhou, China; ^2^Co-innovation Center for Modern Production Technology of Grain Crops of Jiangsu Province/Key Laboratory of Crop Genetics and Physiology of Jiangsu Province, Yangzhou University, Yangzhou, China; ^3^College of Bioscience and Biotechnology, Yangzhou University, Yangzhou, China; ^4^Root Biology Center, Fujian Agriculture and Forestry University, Fuzhou, China; ^5^Ministry of Education Key Laboratory of Molecular and Cellular Biology, Hebei Collaboration Innovation Center for Cell Signaling and Environmental Adaptation, Hebei Key Laboratory of Molecular and Cellular Biology, College of Life Sciences, Hebei Normal University, Shijiazhuang, China; ^6^Joint International Research Laboratory of Agriculture and Agri-Product Safety, Institutes of Agricultural Science and Technology Development, Yangzhou University, The Ministry of Education of China, Yangzhou, China

**Keywords:** *Oryza sativa* L., natural accessions, salt tolerance, developmental stages, genome-wide association study (GWAS)

## Abstract

Salinity is one of the main adverse environmental factors severely inhibiting rice growth and decreasing grain productivity. Developing rice varieties with salt tolerance (ST) is one of the most economical approaches to cope with salinity stress. In this study, the salt tolerance of 220 rice accessions from rice diversity panel l (RDP1), representing five subpopulations, were evaluated based on 16 ST indices at both seedling and reproductive stages under salt stress. An apparent inconsistency was found for ST between the two stages. Through a gene-based/tightly linked genome-wide association study with 201,332 single nucleotide polymorphisms (SNPs) located within genes and their flanking regions were used, a total of 214 SNPs related to 251 genes, significantly associated with 16 ST-related indices, were detected at both stages. Eighty-two SNPs with low frequency favorable (LFF) alleles in the population were proposed to hold high breeding potential in improving rice ST. Fifty-four rice accessions collectively containing all these LFF alleles were identified as donors of these alleles. Through the integration of meta-quantitative trait locus (QTL) for ST and the response patterns of differential expression genes to salt stress, thirty-eight candidate genes were suggested to be involved in the regulation of rice ST. In total, the present study provides valuable information for further characterizing ST-related genes and for breeding ST varieties across whole developmental stages through marker-assisted selection (MAS).

## Introduction

Rice (*Oryza Sativa* L.) is one of the most important food crops that feeds more than half of the population of the world. It is estimated that until 2050, the population of the world will reach 9 billion, which poses a great challenge for crop productivity including rice production (Tyczewska et al., 2018). Improving rice tolerance to various biotic and abiotic stresses is an important strategy to increase rice yield. Salinity stress, as one of the main adverse environmental factors, inhibits plant growth and decreases crop productivity. Globally, more than 930 million hectares of land are reportedly affected by salinity (Hu et al., 2012). In addition, irrational use and management of water and fertilizer may speed up soil salinization, which has been found in some areas especially in rice growing areas (Yamaguchi and Blumwald, 2005). Known as a glycophyte, very few rice varieties could grow on highly salinized land, and rice in seedling and reproductive stages is generally most sensitive to salt stress (Khan et al., 2008; Ali et al., 2014; Emon et al., 2015; Rohila et al., 2019). Excess salt can interfere with metabolic processes at multiple levels, resulting in a reduction of the seed germination rate and interruption of the normal growth and the ultimate grain yield losses (Zeng and Shannon, 2000; Khan et al., 2008). Developing rice varieties with salt tolerance (ST) is considered as the most promising, less resource-consuming, and socially acceptable approach to cope with salinity stress and to take full advantage of marginal lands.

Rice varieties vary drastically on ST-associated characteristics, which provides the basis to develop new varieties with improved ST and high yield. In general, rice ST is a comprehensive expression of a variety of physiological responses of rice under salt stress, typically known as a quantitative trait loci (QTLs) controlled by multiple genes (Khan et al., 2008; Ganie et al., 2019). In the past two decades, dozens of ST QTLs and/or genes and their linked markers have been identified, some of which have already been successfully incorporated into commercial cultivars (Emon et al., 2015; Hossain et al., 2015; Ganie et al., 2019). A classic example is Saltol, a QTL with major effects on ST mapped on chromosome 1 (Bonilla et al., 2002), in which *SKC1* was firstly cloned as the ST-related gene encoding Na^+^-selective transporter (Ren et al., 2005). *SKC1* plays an important role in maintaining Na^+^/K^+^ homeostasis, thereby contributing profoundly to ST (Mohammadi-Nejad et al., 2008; Thomson et al., 2010; Ganie et al., 2016). Due to the minor effects, however, most of ST QTLs were in fact neither isolated nor characterized, which limited their utilization in breeding programs. Through marker assisted selection (MAS), the *SKC1* had been incorporated into some rice varieties with apparently enhanced ST in the seedling stage. It has been well recognized that ST at seedling and reproductive stages are different (Moradi et al., 2003; Liu et al., 2019; Chen et al., 2020; Lei et al., 2020), while only few studies have reported ST QTLs/genes at the reproductive phase so far (Mohammadi et al., 2013; Bimpong et al., 2014; Hossain et al., 2015; Ganie et al., 2019; Lekklar et al., 2019; Liu et al., 2019; Theerawitaya et al., 2020). Through the linkage mapping method, Hossain et al. (2015) reported 16 QTLs affecting 6 agronomic traits, plus Na^+^ content, and the K^+^/Na^+^ ratio in the flag leaf under stress conditions at the reproductive stage. In recent years, genome-wide association study (GWAS) has been developed in crops to enable large-scale and high-precision identification of elite alleles, and their allelic variations widely distributed in natural rice varieties (Zhao et al., 2011; Lekklar et al., 2019; Liu et al., 2019). With respect to salt stress, Kumar et al. (2015) firstly conducted GWAS with high density single nucleotide polymorphism (SNP) markers and identified 64 SNPs significantly associated with the K^+^/Na^+^ ratio and some agronomic traits under salt stress at rice reproductive stage. Lekklar et al. (2019) investigated photosynthetic and yield-related traits of 190 Thai and Asian rice accessions exposed to salt stress at the flowering stage and identified 448 SNPs associated with ST by GWAS. By using 708 rice accessions and high-density SNPs within genes, Liu et al. (2019) detected 2,255 SNPs that were significantly associated with ST-related traits at both seedling stage in greenhouse and reproductive stage at saline fields. In total, although a lot of ST-related QTLs/genes have been reported, few of them, especially for those at reproductive stage, were characterized and successfully used in breeding (Li et al., 2020; Theerawitaya et al., 2020; Nayyeripasand et al., 2021).

Besides high-density SNPs generally used in GWAS, the other advantage of GWAS is that once a natural variety population is genotyped, it can be widely used in different studies toward diverse traits. For instance, to the best of our knowledge, the rice diversity panel l (RDP1), comprised around 420 varieties from 82 countries/regions and genotyped by high-density SNP markers, has been widely employed in studying agronomic traits and biotic and abiotic stresses (Famoso et al., 2011; Zhao et al., 2011; Norton et al., 2014; Kang et al., 2016; McCouch et al., 2016; Chen et al., 2019; Feng et al., 2019). However, although the GWAS using natural variety population has a few advantages than QTL mapping based on bi-parents population on detecting ST-related QTLs, as one of forward genetic approaches, it is still very difficult on identifying genes that underly these ST QTLs. Comparatively, most ST-related genes reported so far were obtained by reverse genetic approach, which significantly deepened the understanding of ST mechanism although the breeding potential of these genes remained to be evaluated (Ganie et al., 2019; Zhang et al., 2021). Notably, genome-wide transcriptional reprogramming reflected by differentially expressed genes (DEGs) response to ST tolerance, such as the genes related to osmotic adjustment, detoxification, repair of stress-induced damage, amplification or attenuation of stress signaling, and so on, has been found play important roles in ST tolerance (Zhang et al., 2021). This implies that identifying ST-related DEGs located in ST QTLs or associated SNPs regions is one of important approaches to quickly screen potential candidate genes or elite alleles underlying these QTLs. In the present study, the ST performance of 220 rice accessions from RDP1, representing five subpopulations (tropic japonica/TRJ, temperate japonica/TEJ, indica/IND, aus/AUS, aromatic/ARO) and an admixture (ADMIX) subpopulation, has been evaluated at both seedling and reproductive stages. By using a gene-based/tightly linked GWAS analysis as reported in Liu et al. (2019), we identified 214 SNPs related to 251 genes that are significantly associated with ST at both stages. Especially, fifty-four rice accessions were proposed as donors with most valuable alleles in breeding and 31 promising candidate ST genes were recommended after the integrated analysis with public DEG data. These results provide useful information to further characterize ST-related genes and for breeding ST varieties using the proposed donors through MAS.

## Results

### Phenotypic Variations at Seedling and Reproductive Stages

**FIGURE 1:**
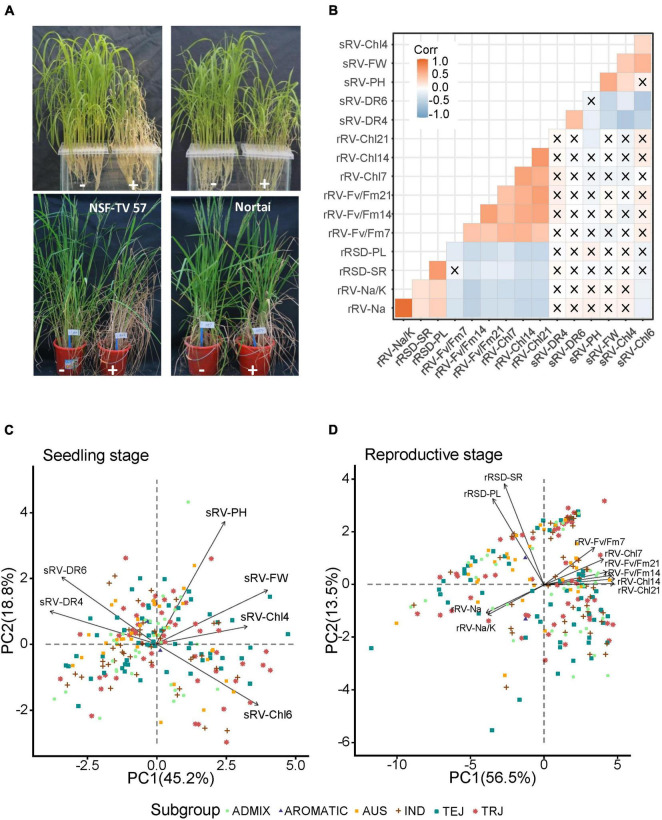
Phenotype and principal component analysis (PCA) of indices under salt stress at seedling and reproductive stages. **(A)** Comparison of rice accessions with (+) and without (–) NaCl treatment. Pictures for seedling stages were taken at 4 days after treated with and without 0.8% NaCl. Pictures for reproductive stages were taken at 14 days after treated with and without 0.5% NaCl. Left and right pictures in each panel refer to different rice accessions with different tolerance to salt stress at seedling or reproductive stages, respectively. **(B)** Pearson correlation coefficient matrix of 16 ST indices. The cross indicates that the correlations did not reach statistically significant level at *p* < 0.05. Red and blue refer to positive and negative correlation, respectively. **(C,D)** PCA analysis of salt tolerance (ST) indices at seedling and reproductive stages, respectively. Total of 220 accessions were plotted on PCA plot with different shapes and colors. Each arrow represents an index and the length refers to its contribution on ST. The angles captained by any of the two arrows less than 90° imply the two indices having positive correlation, otherwise represent negative correlation between the two indices.

At the seedling stage, we found that most accessions began to wither and turn yellow on the 4th day after salt treatment and died on the 6th day after salt treatment, while a few accessions still showed acceptable growing status on the 6th days after salt treatment (Figure 1A and Supplementary Figure 1). According to the phenotypic distribution, we found that sRV-Chl6 and sRV-DR6 presented an apparently skewed distribution, while the rest four ST-related indices displayed a more scattered distribution (Supplementary Figure 1), demonstrating a significant variance of ST among accessions tested at the seedling stage.

At the reproductive stage, ten ST-related indices were scored (Supplementary Table 1). Na^+^ content and Na^+^/K^+^ ratio, which are commonly important indices in evaluating crop ST, clearly showed scattered distributions in the population, demonstrating varying responses to salt stress among accessions (Supplementary Figure 2). Similar trends were observed in 6 indices related to chlorophyll contents and Fv/Fm. Some accessions were severely affected by salt treatment, showing no panicle emergence, while some others exhibited apparently reduced panicle lengths (Figure 1A). Therefore, the panicle length was measured as one of agronomical indices. We found that compared with the control, only a few accessions produced similar panicle lengths under salt treatment conditions, and most had significantly shortened panicles, including some without panicles (Supplementary Figure 2). The other agronomic index is seed-setting ratio (rRSD-SR), which showed a clearly scattered distribution in the population. Together, these data indicate that the accessions in this study display significant differences in the ST at both seedling and reproductive development stages.

Correlation analysis showed that almost no significant correlations were found between ST indices from seedling stage and reproductive stage (Figure 1B). This is consistent with previous studies, in which very few ST-related QTLs were reported conferring ST at both seedling and reproductive stages (Hossain et al., 2015; Liu et al., 2019). In addition, we also conducted a multi-comparison of each ST index among 5 subpopulations, and only observed three traits that showed significant differences among some subpopulations (Supplementary Figure 3). One index was sRV-FW that significantly differed between ADMIX and TRJ subpopulations. The other two were rRSD-SR and rRV-Fv/Fm7, which showed significant differences between AUS and TEJ subpopulations, and between AUS subpopulation and IND and TRJ subpopulations, respectively. This implies that the five subpopulations have almost no differentiation on ST.

In order to better portray the ST performance of the accessions, we conducted principal component analysis (PCA) for all measured ST-related traits. For the seedling stage, the PCA results showed that PC1 accounted for more than 45.2% of the total variance while PC2 was responsible for 18.8% (Figure 1C). According to the length of each index line on PCA plot, the sRV-PH presented the strongest correlation with ST, followed by sRV-FW, sRV-Chl6, sRV-DR6, sRV-DR4, and sRV-Chl4. Negative correlations between photosynthesis and survival rates under stress have been well documented (Moradi and Ismail, 2007), consistent with our data that sRV-Chl4 and sRV-Chl6 were significantly negatively correlated with the sRV-DR4 and sRV-DR6, respectively, reflected by their opposite directions on the PCA plot. For the reproductive stage, the PCA results showed that PC1 and PC2 accounted for 56.5% and 13.5% of the total variance, respectively (Figure 1D). Comparatively, the two agronomic traits, rRSD-SR and rRSD-PL, directly associated with grain yield and showed strong correlations with ST, while other indices presented similarly moderate contributions to ST, according to the PCA analysis. The Na^+^ content and Na^+^/K^+^ ratio, commonly used for evaluating ST in seedlings (Zelm et al., 2020), did not appear to be strongly correlated with ST at the reproductive stage. Except for the correlation between rRSD-SR and rRV-Fv/Fm7, all other correlations among 10 indices reached a statistically significant level (Figure 1B). Both Na^+^ content and Na^+^/K^+^ ratio showed apparently negative correlations with the rRV-Chl and rRV-Fv/Fm indices, while positive correlations were identified with the two agronomic traits, rRSD-PL and rRSD-SR, indicating that a higher content of Na^+^ leads to a more severe hurt to the plants (Figure 1B). Together, the PCA data indicate that different indices have divergent contributions to rice ST at different stages.

### Genome-Wide Association Study of Salt Tolerance-Related Indices

To elucidate the genetic variance and identify the potential gene(s) with ST at both seedling and reproductive stages, we conducted a gene-based/tightly linked GWAS for 16 ST-related indices. A total of 201,332 SNPs located within genes and their flanking regions were identified, which related to 27,923 unique genes (Supplementary Table 2). The average interval distance of these SNPs on genome is 1,892 bp and ranges from 1,543 to 2,225 bp on different chromosomes (Chrs), showing a relatively even distribution on rice genome (Supplementary Figure 4). By using two statistical analysis models, Compressed Mixed Linear Model (CMLM) and Bayesian-information and Linkage-disequilibrium Iteratively Nested Keyway (BLINK), we obtained a total of 252 and 508 SNPs significantly associated with ST, respectively, which covered 15 ST-related indices except for sRV-DR4 on which no significant SNP was detected (Supplementary Tables 3–18). Comparatively, a total of 224 SNPs were consistently detected by both methods (Supplementary Table 19). After removing 10 SNPs that were repeatedly detected in different ST indices, 214 SNPs in total were chosen for further analysis.

**FIGURE 2:**
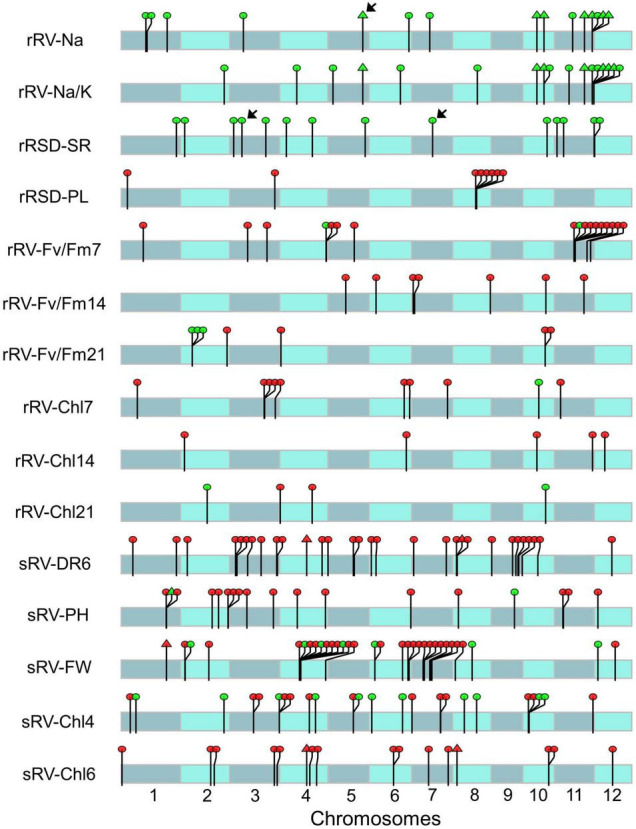
Distribution of significant single nucleotide polymorphisms (SNPs) associated with different ST indices on chromosomes. Red and green dots indicate the corresponding SNP explained phenotypic variation less than 10% and more than 10%, respectively. The arrows indicate the SNPs explain more than 14% total phenotypic variation of the corresponding ST index. Triangles indicate the SNPs associated with two different ST indices. The length of chromosome was dimensionally drawn based on the physical length of each chromosome (http://rice.plantbiology.msu.edu).

A total of 117 significant SNPs were detected at the seedling stage, which were distributed on different chromosomes and corresponded to 140 genes but varied among traits (Figure 2 and Supplementary Table 19). Specifically, 32 SNPs representing 39 genes for sRV-FW were identified on Chrs 1, 2, 4, 6, 7, 8, and 12, which accounted for 8.26- 12.31% of phenotypic variance. For sRV-DR6, 30 SNPs from 37 genes were found on the genome except for Chr 11, with a notable preference for Chr 3 and Chr 9, each harboring 7 and 9 SNPs, respectively. For sRV-Chl6, 16 SNPs belonging to 21 genes were evenly located on Chrs 1, 2, 3, 4, 6, 7, 8, 10, and 12, while the associated SNPs for sRV-Chl4 were preferentially situated on Chr 3 (5 out of 24) and Chr 10 (4 out of 24), with phenotypic variance ranging from 8.65 to 11.52%. For sRV-PH, the 18 SNPs from 21 genes were discovered on Chrs 1, 2, 3, 4, 6, 8, 9, 11, and 12, accounting for 8.79-10.20% of phenotypic variance. Out of the above associations, three SNPs were linked with two traits: one of them (SNP-1.33074009) was detected in both sRV-FW and sRV-PH, while the other two SNPs (SNP-4.19825069 and SNP-8.3430353) were consistently found in sRV-DR6 and sRV-Chl6, possibly due to pleiotropic effects of the SNP-carrying genes.

At the reproductive stage, a total of 97 significant SNPs corresponding to 111 genes interspersed on the genome were detected with 17 for rRV-Fv/Fm7, 17 for rRV-Na/K, 15 for rRV-Na, 14 for rRSD-SR, 10 for rRV-Chl7, 8 for rRSD-PL, 7 for rRV-Fv/Fm14, 7 for rRV-Fv/Fm21, 5 for rRV-Chl14, and 5 for rRV-Chl21, respectively (Supplementary Table 19). The phenotypic variance of these significant associations ranged from 5.62 to 16.90%. Around 25% (25 out of 97) of SNPs were detected on Chr 11, including 10 for rRV-Fv/Fm7, 8 for rRV-Na/K, 6 for rRV-Na, 2 for rRSD-SR, and 1 for each of rRV-Fv/Fm14, rRV-Chl7, and rRV-Chl14. On Chr12, only 3 associations were found, 2 for rRSD-SR and 1 for rRV-Chl14. The rest of the ST-related signals were distributed among the other Chrs except Chr 9, each containing 5-12 SNPs. While 7 SNPs were jointly associated with Na^+^ and Na^+^/K^+^, which are highly correlated (*r* = 0.94, Figure 1B), none of the evaluated index-associated SNPs/genes at this stage overlapped with those detected at the seedling stage, further revealing the complexity and stage specificity of rice ST.

### Pyramiding Effects of Low-Frequency Favorable Single Nucleotide Polymorphism-Carrying Genes

To identify favorable alleles/haplotypes with potential effects on ST improvement, we conducted a haplotype analysis for each SNP locus (Supplementary Table 19). According to the result of *t*-test between the two genotypes in each SNP locus, we found that 172 SNPs related to 201 genes exhibited significant differences on corresponding ST index value, and the frequency for the favorable alleles ranged from 2.80 to 97.18% in the population (Supplementary Table 19). In particular, we are interested in those favorable alleles less than 50% in the population and consider them as low-frequency favorable (LFF) alleles, because they are not utilized in most varieties and so have a relatively high breeding value in ST improvement for most varieties. Certainly, those high-frequency favorable (HFF) alleles (> 50% in the population) are also helpful to improve some varieties without them. After screening, a total of 82 LFF alleles (refer to 96 genes) with the frequency ranging from 2.80 to 50% were shortlisted, which included 57 and 25 (67 and 29 genes) at the seedling and the reproductive stages (Supplementary Table 20), respectively.

**FIGURE 3:**
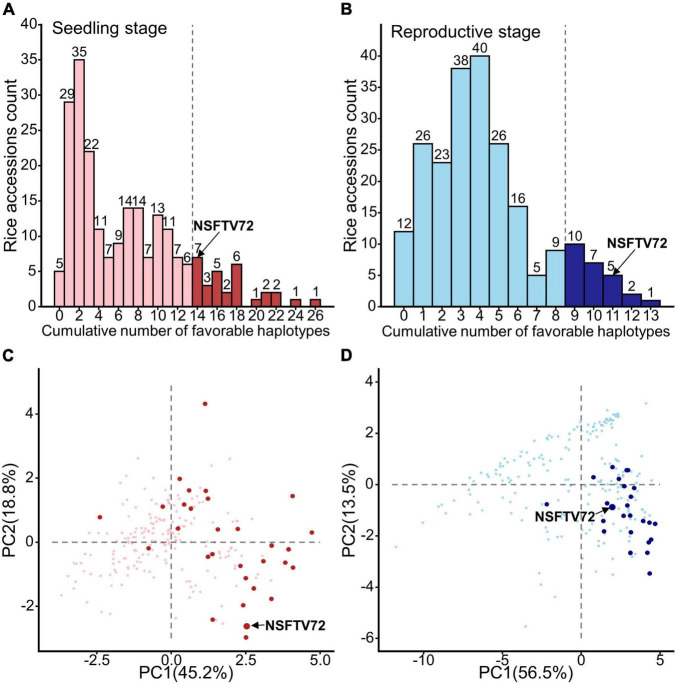
Distribution of accessions contained low frequency favorable alleles and the potential donor germplasms of these alleles. **(A,B)** The histograms for the count of rice accessions with cumulative number of favorable haplotypes at seedling **(A)** and reproductive **(B)** stages. The dark columns at the right of dotted line represent the top 15% accessions that carrying most low-frequency favorable (LFF) alleles at seedling and reproductive stages, respectively. Number on each column indicates the number of accessions containing the corresponding number of LFF alleles. **(C,D)** Selected donor germplasms on the PCA plots similar to Figure 1 **C,D**. Dark points in panels **(C,D)** represent the top 15% accessions that carried most LFF alleles at seedling and reproductive stages, respectively. NSFTV72 is IR8, containing 14 and 11 LFF alleles at seedling and reproductive stages, respectively.

To assess whether these LFF alleles have pyramiding effects on ST improvement, we evaluated the relations between the ST index value and the number of LFF alleles. As shown in Supplementary Figures 5, 6, an apparently linear relationship was observed between each of 16 ST index values and the number of LFF alleles, although the degrees of slopes varied among each other that reflect the magnitude of effects. These additive effects inform that the adaptability of rice to salinity can be strengthened by combining LFF alleles that dominate ST-related indices. Based on this consideration, we then calculated the number of stage-specific LFF alleles accumulated in each accession. At the seedling stage, we found that no varieties contained more than half of the total LFF alleles, and the highest number of LFF alleles accumulated in a variety was 26 (Figure 3A). Most varieties harbored less than 10 LFF alleles and around 46% (number of 102) varieties possessed no more than 4 LFF alleles. At the reproductive stage, one variety carried the highest number of 13 LFF alleles, and up to 63% germplasms (139) contained less than 4 LFF alleles (Figure 3B).

Considering the fact that LFF alleles are distributed in different germplasms, we selected the accessions that possessed top 15% stage-specific LFF alleles as promising germplasms at each developmental stage. At the seedling stage, 30 accessions carrying LFF alleles with numbers ranging from 14 to 26 were identified as potential ST breed donors (Figure 3A), which covered all 57 LFF alleles (Supplementary Table 21). Among them, most accessions contained 14 to 18 LFF alleles, and 7 accessions possessed more than 20 LFF alleles. At the reproductive stage, 25 accessions carrying at least 9 LFF alleles were identified as potential ST breed donors with 3 of them possessing more than 12 LFF alleles (Figure 3B). Together, these 25 accessions covered all 25 reproductive specific LFF alleles (Supplementary Table 22). In order to better present the ST performance of these potential donors, we projected them on the PCA plot (Figures 3C,D). According to the directions of each ST index on the PCA plot (Figures 1C,D), we knew that the accessions located in the right part had better ST performance. At the seedling stage, we found that except for 3 accessions, all the rest 27 varieties were distributed in the right part of PCA plot (Figure 3C). With respect to the reproductive stage, only one variety was located at the left part of the PCA plot and over 76% (19/25) of the varieties were distributed at the bottom right section of PCA plot, suggesting that these varieties have good ST performance at the reproductive stage (Figure 3D). Normally, rice ST acquired at the seedling stage does not sustain through the reproductive stage (Singh and Flowers, 2010). Yet, we found an accession (NSFTV72/IR8) that featured a longer life span in ST, although it did not display the best ST at each of the two stages (Figures 3C,D). This variety accumulated 14 seedling LFF and 11 reproductive LFF alleles (Figures 3A,B), suggesting its potential value as a donor for simultaneously transferring multiple LFF alleles from both stages in future breeding programs.

### Candidate Genes Associated With Salt Tolerance-Related Indices

Since all the SNPs applied in the above GWAS analysis are located in or very close to the annotated genes, we would like to mine some candidate genes for ST. Out of 214 significant SNPs, we found that 39 SNPs were located in the coding regions (Supplementary Table 23). These included 17 synonymous SNPs and 22 non-synonymous SNPs resulting in either amino acid substitutions or premature stop codons. The rest of the 175 SNPs were in non-coding regions, which can be further subdivided into three groups: 127 in intergenic regions, 5 in 5′ UTR, 7 in 3′ UTR, and 36 in intron (Supplementary Table 23). Altogether, these 214 significant SNPs cover 251 genes, in which 222 are annotated and 29 encoded hypothetical proteins of unknown functions (Supplementary Table 19).

**FIGURE 4:**
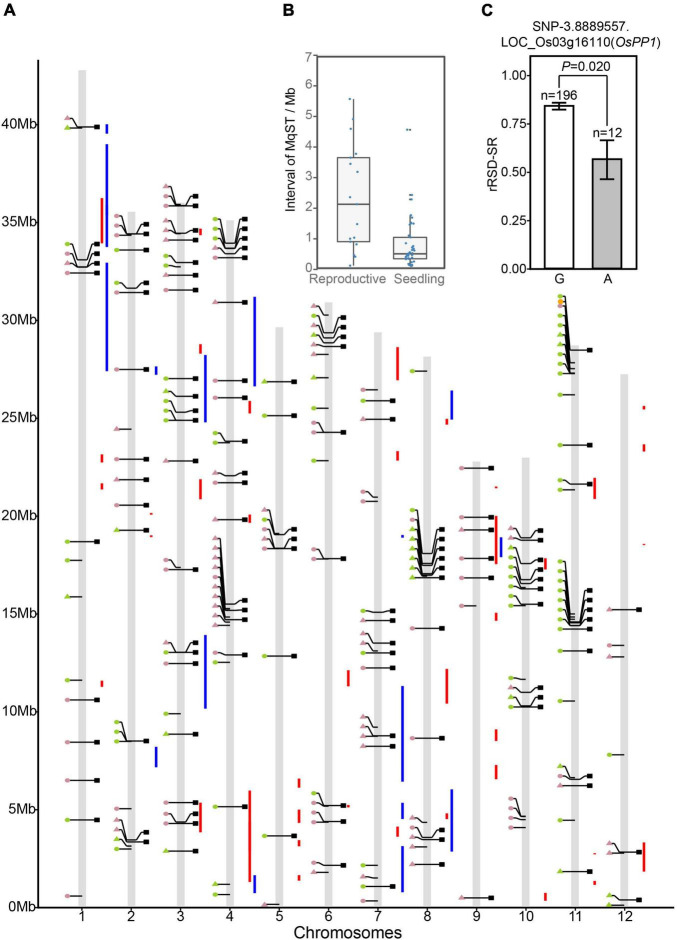
Comparison of the significant association SNPs, Meta-quantitative trait locus (QTL), and differentially expressed genes. **(A)** Integrated map for association SNPs, Meta-QTL intervals and differentially expressed genes (DEGs). Circles and triangles on the left side of each chromosome represent LFF and not LFF SNPs, respectively. Green and pink colors indicate the association SNPs detected at seedling and reproductive stages, respectively. Squares on the right side of the chromosomes represent the DEGs. Red and blue vertical lines refer to meta-QTL intervals of seedling and reproductive stages, respectively. **(B)** Distribution of interval length of meta-QTL in reproductive stage (rMqST) and in seedling stage (sMqST). **(C)** Haplotype analysis of the SNP-3.8889557 associated differentially expressed gene *OsPP1*. The *n* = 196 and *n* = 12 indicate the number of the accessions harbored “G” and “A” haplotypes, respectively. The average rRSD-SR value of the accessions containing each of the two haplotypes reached statistically significant difference at *p* < 0.05.

To prioritize GWAS-derived SNPs/genes, we conducted a co-localization analysis with ST-related QTLs published previously. Since most of these reported QTL data came from independent studies that were not entirely consistent with each other, we performed a meta-analysis to identify consensus QTLs with high reliability. A sum of 375 ST-related QTLs from 18 independent studies were collected for meta-analysis, which included 308 in the seedling stage, 55 in the reproductive stage, and 12 in vegetative and reproductive stages (Supplementary Table 24). In meta-analysis, QTLs in vegetative and reproductive stages were all considered as reproductive ST QTL. As a result, we obtained 41 seedling meta-QTL for ST (sMqST) and 17 reproductive meta-QTL for ST (rMqST), which were scattered on all 12 chromosomes (Figure 4A and Supplementary Table 25). The interval length of these sMqST and rMqST ranged from 0.054 to 4.734 Mb and from 0.139 to 5.614 Mb, respectively (Supplementary Table 25). The average length of sMqST was 0.789 Mb and apparently less than 2.375 Mb of rMqST (Figure 4B). Through comparison, we found a total of 45 SNPs located in these MqST intervals (Figure 4A).

To screen candidate genes for ST, we consulted publicly available data on DEGs of rice under salt stress at diverse growth stages, which offered a list of 143 DEGs that related to 129 out of 214 SNPs (Supplementary Table 26). To the best of our knowledge, only one of them, *OsPP1* (LOC_Os03g16110), encoding a protein phosphatase, has been reported as an ST regulation gene (Liao et al., 2016). Rice plants over-expressing *OsPP1a* can recover from salinity-induced oxidative damage via boosting antioxidant enzyme systems to maintain a relative redox homeostasis. SNP-3.8889557, associated with rRSD-SR on chromosome 3, was found to be located in the 3′ UTR of *OsPP1*, which accounted for 14.48% of the total variation of rRSD-SR (Supplementary Table 23). Twelve accessions out of 208 (5.77%) carried the “A” allele at this position with an average rRSD-SR of 56%, while the accessions possessing the alternative “G” allele were more sensitive to salinity treatment, with an average rRSD-SR of 84% (Figure 4C). In comparison, the favorable “A” allele of *OsPP1* was able to rescue 28% losses on seed setting rate due to salt stress during the reproductive stage. This implies that mining DEG data could be a feasible strategy to screen candidate ST genes.

**FIGURE 5:**
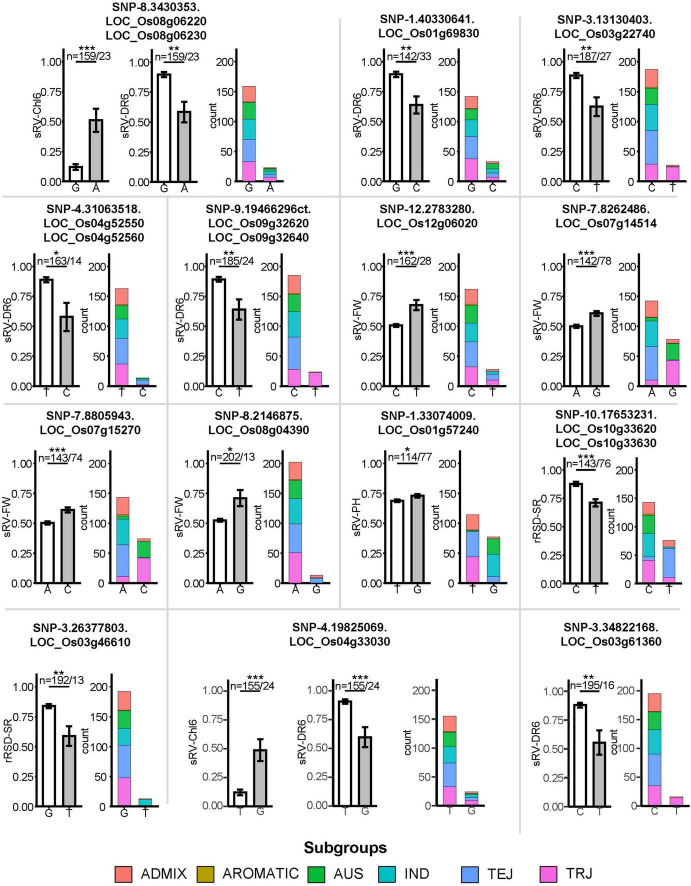
Effects comparison of different haplotypes in 14 LFF SNPs associating with differentially expressed genes on salt tolerance. Error bars mean the standard errors. *, ** and *** indicate the difference reached statistically significant difference at the *P* value <0.05, <0.01, and <0.001, respectively. Numbers of rice accessions contained different alleles in each SNP locus were listed on the top of columns. Two SNPs were found association with two ST indices.

By comparison, we identified 38 DEGs related to 32 out of 45 SNPs located within MqST intervals (Table 1). The chromosome 3 had most co-located DEGs with MqST, and no co-located DEGs and MqST were found on chromosome 5 (Figure 4A). According to the gene annotation, we found that except for 4 genes belonging to hypothetical protein or predicted protein, most of the rest of the 34 DEGs presented potential function in various kinds of stress resistance, such as *OsPRI1*, *OsSPL2*, *OsULT1*, *OsERF60*, and so on (Table 1). It is worth further validation on the function of these candidates in ST. In addition, eighteen of these candidates were from LFF alleles, which related to 5 ST indices with 3 genes for sRV-Chl6, 10 for sRV-DR6, 4 for sRV-FW, 1 for sRV-PH, and 3 for rRSD-SR (Figure 5). To estimate the contribution of each LFF of these 18 candidates, we compared the differences of accessions with favorable and unfavorable alleles on the corresponding ST indices and found that all favorable alleles could significantly improve the ST tolerance reflected by the corresponding ST indices (Figure 5). Certainly, the exact contributions on ST improvement of these LFF alleles required to be tested in practice in the future. Through qRT-PCR, we randomly tested transcription levels of 6 candidates from LFF alleles and one gene *OsPP1*(LOC_Os03g16110) in 15 accessions at 12 and 24 h after treatment (HAT) with salt (Supplementary Figure 7). Both favorable and unfavorable alleles in each of these 7 genes were included in the 15 accessions (Supplementary Table 27). We found that the transcriptional levels of all 7 genes were significantly induced by salt stress, which demonstrated not only their potential roles in regulating rice ST but also the reliability of other candidate genes in Table 1. Among them, *OsPRI1* (LOC_Os08g04390) and *OsULT1* (LOC_Os01g57240) showed that differently induced expression levels by salt stress between the accessions contained favorable and unfavorable alleles, while the remaining 5 genes did not show this difference.

**TABLE 1:** Details of candidate genes for salt tolerance and its co-located single nucleotide polymorphisms (SNPs) and meta quantitative ST loci (MqSTs).

MSU.ID	Chr	SNP	Trait	MqST	GSE.ID	LFF	Description
LOC_Os01g57240	1	SNP-1.33074009.	sRV-PH,sRV-FW	rMqST1-1	GSE21651,GSE16108,GSE6901	yes	OsULT1,SAND domain-containing protein, Trithorax group factor, Transcriptional regulation of stress responsive genes
LOC_Os01g69830	1	SNP-1.40330641.	sRV-DR6	rMqST1-4	GSE21651,GSE16108,GSE6901	yes	OsSPL2, Squamosa promoter-binding-like protein 2;Similar to SBP-domain protein 4
LOC_Os03g22740	3	SNP-3.13130403.	sRV-DR6	rMqST3-1	GSE21651	yes	BIP102, Brassinosteroid receptor kinase-interacting protein 102;Similar to SAR DNA-binding protein-like protein
LOC_Os03g46610	3	SNP-3.26377803.	rRSD-SR	rMqST3-2	GSE21651	yes	TOGR1, DDX47, OsRH10, Thermotolerant growth required 1, ATP-dependent RNA helicase DDX47, RNA helicase 10
LOC_Os03g61360	3	SNP-3.34822168.	sRV-DR6	sMqST3-4	GSE16108,GSE6901	yes	Similar to Soluble epoxide hydrolase
LOC_Os04g33030	4	SNP-4.19825069.	sRV-Chl6,sRV-DR6	sMqST4-3	GSE21651	yes	SIP23, Zinc finger RING-type domain containing protein
LOC_Os04g52550	4	SNP-4.31063518.	sRV-DR6	rMqST4-2	GSE21651,GSE16108	yes	OsAGO3, Protein argonaute 3-like
LOC_Os04g52560	4	SNP-4.31063518.	sRV-DR6	rMqST4-2	GSE21651,GSE16108	yes	FAR1 domain containing protein
LOC_Os07g14514	7	SNP-7.8262486.	sRV-FW	rMqST7-3	GSE21651	yes	Similar to OSIGBa0140C02.4 protein
LOC_Os07g15270	7	SNP-7.8805943.	sRV-FW	rMqST7-3	GSE16108,GSE21651	yes	RNA recognition motif, RNP-1 domain containing protein
LOC_Os08g04390	8	SNP-8.2146875.	sRV-FW	sMqST8-1	GSE21651,GSE16108	yes	OsPRI1, bHLH transcription factor, Positive regulator of iron homeostasis 1
LOC_Os08g06220	8	SNP-8.3430353.	sRV-Chl6,sRV-DR6	rMqST8-1	GSE21651	yes	Transferase domain containing protein
LOC_Os08g06230	8	SNP-8.3430353.	sRV-Chl6,sRV-DR6	rMqST8-1	GSE21651,GSE6901	yes	GTP1/OBG domain containing protein
LOC_Os09g32620	9	SNP-9.19466296ct.	sRV-DR6	sMqST9-4	GSE16108,GSE21651	yes	Alcohol dehydrogenase superfamily, zinc-containing protein
LOC_Os09g32640	9	SNP-9.19466296ct.	sRV-DR6	sMqST9-4	GSE21651	yes	Similar to Quinone-oxidoreductase QR1 (Fragment)
LOC_Os10g33620	10	SNP-10.17653231.	rRSD-SR	sMqST10-2	GSE21651	yes	Ubiquitin domain containing protein
LOC_Os10g33630	10	SNP-10.17653231.	rRSD-SR	sMqST10-2	GSE21651	yes	OsABCI16, Adaptin ear-binding coat-associated protein 1 NECAP-1 family protein
LOC_Os12g06020	12	SNP-12.2783280.	sRV-FW	sMqST12-1	GSE21651,GSE16108	yes	Dcp1-like decapping family protein
LOC_Os01g56790	1	SNP-1.32769262.	sRV-PH	rMqST1-1	GSE21651,GSE16108	no	Conserved hypothetical protein
LOC_Os01g57260	1	SNP-1.33085898.	sRV-PH	rMqST1-1	GSE21651,GSE16108	no	Vacuolar protein sorting-associated, VPS28 family protein
LOC_Os02g45660	2	SNP-2.27769525.	sRV-PH	rMqST2-2	GSE21651	no	OsIspF, 2-C-methyl-d-erythritol 2,4-cyclodiphosphate synthase
LOC_Os03g08360	3	SNP-3.4267308.	sRV-DR6	sMqST3-1	GSE21651	no	OsONI1, Fatty acid elongase (beta-ketoacyl-CoA synthase), Shoot development
LOC_Os03g08460	3	SNP-3.4343151.	sRV-DR6	sMqST3-1	GSE21651,GSE16108	no	OsERF60, AP2/EREBP27, OsEBP89; APETALA2/ethylene responsive factor, ERF transcription factor, Tolerance to drought and submergence stress
LOC_Os03g21950	3	SNP-3.12546468.	sRV-PH	rMqST3-1	GSE21651,GSE16108	no	Predicted protein
LOC_Os03g22730	3	SNP-3.13123923.	rRV-Fv/Fm7	rMqST3-1	GSE21651,GSE6901	no	BIP101, brassinosteroid receptor kinase (BRI1)-interacting protein 101
LOC_Os03g44660	3	SNP-3.25139625.	rRV-Chl7	rMqST3-2	GSE21651,GSE3053	no	OsGRL7, GRX-like protein 7, glutaredoxin-like protein 7; Thioredoxin fold domain containing protein
LOC_Os03g45280	3	SNP-3.25559362.	rRV-Fv/Fm7	rMqST3-2	GSE16108	no	OsLEA24, Similar to LIP5
LOC_Os03g48020	3	SNP-3.27296133.	rRV-Fv/Fm7	rMqST3-2	GSE21651,GSE4438,GSE6901	no	Conserved hypothetical protein
LOC_Os03g48030	3	SNP-3.27296133.	rRV-Fv/Fm7	rMqST3-2	GSE21651,GSE16108	no	HPP family protein
LOC_Os04g09570	4	SNP-4.5130296.	rRSD-SR	sMqST4-1	GSE16108,GSE6901	no	Conserved hypothetical protein
LOC_Os04g45910	4	SNP-4.27009828.	sRV-Chl6	rMqST4-2	GSE21651	no	Similar to endonuclease, polyU-specific
LOC_Os04g45920	4	SNP-4.27009828.	sRV-Chl6	rMqST4-2	GSE21651	no	OsRLCK155, Receptor-like Cytoplasmic Kinase 155
LOC_Os06g10160	6	SNP-6.5195014.	rRV-Fv/Fm14	sMqST6-1	GSE21651,GSE16108	no	OsRLCK203, Serine/threonine protein kinase domain containing protein
LOC_Os07g02760	7	SNP-7.1005736.	rRV-Fv/Fm14	rMqST7-1	GSE21651,GSE16108	no	OsFbox330, F-box domain, Skp2-like domain containing protein
LOC_Os08g06380	8	SNP-8.3544780.	sRV-DR6	rMqST8-1	GSE21651,GSE4438	no	OsCslF6, MLG (mixed-linkage glucan) synthase, Biosynthesis of MLG (cell wall polysaccharide); Similar to Cellulose synthase-like CslF6
LOC_Os09g29584	9	SNP-9.17990633.	sRV-DR6	sMqST9-4	GSE13735,GSE21651,GSE4438	no	OsWAK84, EGF-like calcium-binding domain containing protein
LOC_Os09g34100	9	SNP-9.20121667.	sRV-DR6	sMqST9-4	GSE21651	no	OsGLYII3, glyoxalase II-3, glyoxalase II
LOC_Os11g37000	11	SNP-11.21374400.	rRV-Na,rRV-Na/K	sMqST11-3	GSE16108	no	OsDjC77, Heat shock protein DnaJ family protein

*MSU.ID: Gene ID in Rice Genome Annotation Project; Chr: chromosome number; GSE.ID: ID in Gene Expression Omnibus Series.*

*OsPRI1*, associated with SNP-8.2146875 in the interval of sMqST8-1, was a more strongly induced expression in 5 accessions that contained favorable alleles than those without favorable alleles at both time-points. In rice, *OsPRI1* is a linker between the iron-binding sensor *OsHRZ1* and the Fe-deficiency-responsive gene *OsIRO2* (Zhang et al., 2017). Under Fe deficiency, this helix-loop-helix (HLH) transcription factor positively regulates *OsIRO2* expression via directly binding to its promotor. Through global bioinformatics analysis, the HLH transcription factor was found to participate in salt responsive gene regulatory networks (Wang et al., 2020). Overexpression of *OsIRO2* results in an elevation of Fe uptake and translocation in rice (Ogo et al., 2007). Being an essential mineral element for plant growth and development, Fe has also been proposed as a defense against salinity since it can boost the production of antioxidative enzymes to mitigate salinity-induced oxidative damage (Sharma et al., 2012; Ghasemia et al., 2014; Li et al., 2016). In addition, salt stress would impose deleterious effects on Fe acquisition and distribution in plants (Li et al., 2016). Consequently, efficient absorption of Fe with the help of *OsIRO2* and its upstream regulator *OsPRI1* would have the potential to confer greater tolerance to saline-alkaline stress in rice. The accessions containing “A” allele in the regulatory of *OsPRI1* displayed a lower sRV-FW of 52%, while only 13 accessions carried the “G” allele that showed a better sRV-FW value (Figure 5). This demonstrated that introducing “G” allele of *OsPRI1* from the 13 accessions could further improve rice ST.

## Discussion

### Identification of Genetic Resources for Rice Breeding Toward Salt Tolerance

Rice is relatively tolerant to salt during germination, active tillering, and maturity, but is very sensitive at the early seedling stage and reproductive stage (Singh and Flowers, 2010; Ganie et al., 2019). To mitigate the adverse effects of salinity on rice, a prospective practice is to breed varieties with salt tolerance at both sensitive stages and develop new rice varieties with enhanced resilience in salt stress environments. However, only a few attempts have been made to discover loci associated with ST across developmental stages and, especially, at the reproductive stage (Ganie et al., 2019; Haque et al., 2020). This is mainly due to the fact that it is so difficult to obtain precise phenotypes at this stage, especially for natural varieties, because they vary largely on the time entering into reproductive stage (Zeng et al., 2002). In order to eliminate the effects of different developing processes on ST-phenotyping, we treated each accession with salt stress at a uniform time, that is, the date of the pulvinus of flag leaf and penultimate leaf reached together. This time point is often defined as rice entering reproductive stage and is easily determined by eye observation (Hoshikawa, 1989; Xu et al., 2020). This design allowed us to establish a uniform standard to test rice ST tolerance at reproductive stage in greenhouse, and to ultimately obtain ST phenotypes as precise as possible for the subsequent analysis. In this study, we evaluated the ST of 220 rice accessions representing five subpopulations at both seedling and reproductive stages by assessing a combination of morphological and physiological parameters. We found that although the degree of ST varies among this rice panel at both stages, almost no correlation was found between ST-related indices measured at seedling stage and reproductive stage (Figure 1B), which is consistent with the previous reports (Moradi et al., 2003; Liu et al., 2019; Chen et al., 2020; Lei et al., 2020). Through GWAS, we identified 117 and 97 SNPs significantly associated with different ST-related indices at seedling and reproductive stages (Figure 2), respectively. Meanwhile, no overlapping SNP was found, highlighting the independence of ST between the two stages and the genetic complexity of rice ST. In addition, we found various correlations among ST indices at the reproductive stage (Supplementary Figure 3), indicating that it is impossible to simply determine critical indices responsible for ST. As a result, we employed PCA to determine the contributions of different indices on ST and found that the two morphological indices (rRSD-PL and rRSD-SR) displayed apparent stronger correlations with ST than those of the other 8 indices, including Na^+^ and Na^+^/K^+^ ratio at the reproductive stage. The rRSD-SR has been universally employed for evaluating rice ST in previous studies, while the rRSD-PL was seldom used. Therefore, the rRSD-PL could be emphasized in both ST-related genetic and breeding research at the reproductive stage in future studies.

The ultimate goal of these significant SNP discoveries is to deploy them in the breeding program to develop new salt-tolerant rice varieties or germplasms that are adaptable to salinity across the whole growth stages. A prospective practice is to combine diverse ST favorable alleles in both sensitive stages of rice. Comparatively, LFF alleles were on the priority list in view of their great potential in genetic improvement. Among the 214 SNPs in total, we identified 57 and 25 LFF alleles at the seedling and the reproductive stages (Supplementary Table 20), respectively. Importantly, our preliminary results showed that rice accessions accumulating more LFF SNPs typically display relatively superior ST compared with those comprising fewer LFF SNPs (Figure 5 and Supplementary Figure 5). This means that increasing the frequency of these LFF alleles in rice population could be a feasible approach to further improve rice ST. In order to accelerate the utilization of these LFF alleles, 30 and 25 potential donors were selected at seedling and reproductive stages, respectively, which collectively covered all 82 LFF alleles and displayed a good performance on ST at both stages (Figure 3). We also noted some deviations where accessions carrying LFF alleles, similar to those of other salt-tolerant lines, exhibit phenotypic salt-sensitivity. This is probably due to the complex interactions among different genes or between genes and genetic backgrounds, which results in various phenotypes, although these accessions harbored similar LFF alleles. Undoubtedly, pyramiding these LFF alleles using the potential donors discovered in this study with previously identified ST loci/genes will facilitate the development of highly tolerant varieties against salinity. In addition, these identified LFF SNPs could also be converted into breeding-friendly and cost-effective markers, like Kompetitive Allele Specific Polymerase Chain Reaction (KASP) marker, for future molecular breeding.

### Candidate Genes Involved in Regulation of Rice Salt Tolerance

In total, 214 significant association SNPs corresponded to 251 genes (Supplementary Table 19). According to a latest review paper on rice ST (Liu et al., 2021), we found that one of the 251 genes was confirmed to be involved in rice ST, which encodes a protein of trehalose family associated with sRV-DR6 in our study (Supplementary Table 28). In addition, we found that 19 known ST-related genes co-located in the LD regions of 19 SNPs (Liu et al., 2021), further confirming the importance of our association study (Supplementary Table 28). By integration of DEG data and Meta-QTL results, we obtained 38 candidate genes that could play a crucial role in tolerance against salinity (Table 1). Seven of them were further confirmed to be induced expression by salt stress, indicating the reliability of these candidate genes in rice ST (Supplementary Figure 7). Two of the 7 genes showed that differently induced expression levels between the accessions contained favorable and unfavorable alleles, which further confirmed the breeding potential of these candidate genes in improving rice ST in practice. In addition, based on the prior literatures, at least 5 genes discussed below were considered having potential roles in ST improvement. All these together imply that these 38 candidate genes are valuable gene resources in further elucidating rice ST.

*OsTOGR1* (LOC_Os03g46610) presents significantly higher expression levels in accessions that contained favorable alleles than those without favorable alleles at 24 hours after treatment (HAT) (Supplementary Figure 7), which encodes a DEAD-box RNA helicase co-located with rMqST3-2. At higher temperature, the intrinsic helicase activity of OsTOGR1 increases, thereby promoting stabilization of pre-rRNA homeostasis (Wang et al., 2016). Although it is uncertain whether *OsTOGR1* directly participates in ST through regulation of RNA surveillance, its two family members, Os*PDH45* and *OsSUV3*, have been demonstrated to function in ST (Amin et al., 2012; Macovei and Tuteja, 2012; Tuteja et al., 2013). In particular, overexpression of *PDH45* confers salt tolerance to rice at both seedling and reproductive stages. SNP-3.26377803, located in the CDS region of *OsTOGR1*, contains two kinds of variations “G” and “T.” Thirteen accessions containing the favorable “T” allele significantly reduced the rRSD-SR of around 25% (Figure 5), implying its breeding potential in ST breeding program.

LOC_Os09g34100 (SNP-9.20121667.), known as *OsGLYII3* and associated with sRV-DR6 at the seedling stage, encodes a Glyoxalase II involved in the glyoxalase pathway (Yadav et al., 2007; Mustafiz et al., 2011), which is required for glutathione-based detoxification of methylglyoxal. Earlier studies have already revealed the potential role of this pathway in conferring ST of tobacco (Singla-Pareek et al., 2003, 2006). Particularly, overexpression of *glyI* from *Brassica juncea* in tobacco imparts tolerance to the transgenic plants during salt stress (Veena et al., 1999); overexpression of *glyI* and *glyII* together can enhance the ST of transgenic plants to a higher extent (Singla-Pareek et al., 2003, 2006). In rice, *OsGLYII3*, together with *OsGLYI1*, were identified as salt responsive gene (Veena et al., 1999; Yadav et al., 2007), with increased transcription levels upon salt stress. Leaf segments from *OsGLYII3*-overexpressing lines could retain a larger amount of chlorophyll in the presence of salt as compared to the control (Singla-Pareek et al., 2008). Consequently, *OsGLYII3* is a prominent causative gene for ST.

LOC_Os02g45660 (SNP-2.27769525.), known as *OsIspF*, is associated with sRV-PH at the seedling stage and encodes a 2-C-methyl-D-erythritol 2,4-cyclodiphosphate synthase, which is the fifth enzyme in the MEP pathway (Huang et al., 2018). Located in chloroplast, *OsIspF* plays a pivotal role in chloroplast development. Rice plants carrying mutated forms of *OsIspF* display yellow-green leaf phenotype throughout development, with significantly reduced contents of chlorophyll and carotenoid. Relationships between photosynthesis, chloroplast, and survival rates of rice under salinity have been well documented (Moradi and Ismail, 2007; Nan et al., 2020). Therefore, *OsIspF* could be a prominent gene for ST.

LOC_Os09g29584 (SNP-9.17990633), also named *OsWAK84*, is associated with sRV-DR6 at the seedling stage and encodes a wall-associated kinase. Wall-associated kinases in rice have been proposed to be involved in plant immunity (Li et al., 2009; Delteil et al., 2016; Harkenrider et al., 2016; Hu et al., 2017) and response to mineral toxicities (Xia et al., 2018). In contrast, there has not been a single report that reveals their relationships with rice ST. However, it was reported that an *Arabidopsis* WAK-like gene 4 (*AtWAKL4*) responded notably to salt stress, with a 5-fold increase in *AtWAKL4* transcripts after salt treatment of the seedlings, while destruction of its promoter via T-DNA insertion extenuated the adaptability of *Arabidopsis* to salt stress (Hou et al., 2005).

LOC_Os03g44660 (SNP-3.25139625), known as *OsGRL7*, is associated with rRV-Chl7 at the reproductive stage and encodes a Glutaredoxins (GRXs) family protein of GRL-type (Garg et al., 2010). Though the precise functions of GRL-type proteins in ST have not been investigated, overexpression of a CPYC-type GRX-OsGRX20 was shown to result in enhanced tolerance to salt stress (Ning et al., 2018). It was proposed that the capacity of GRXs to reduce oxidized disulfides is a decisive factor to maintain redox homeostasis under high salinity (Rouhier et al., 2005; Ning et al., 2018).

The other candidate ST genes jointly supported by DEG data and Meta-QTL results have hardly been studied in the previous literature. In summary, our findings are anticipated to add potential novel gene resource to the list of ST contributing factors. Further functional tests will be needed to validate their roles in ST.

## Materials and Methods

### Plant Materials

A total of 220 rice accessions including 5 subpopulations from RDP1 were selected, and comprised 53 TRJ, 57 TEJ, 43 IND, 32 AUS, 2 ARO, and 33 ADMIX accessions according to that reported in McCouch et al. (2016) (Supplementary Table 1). Twenty germinated seeds of each variety were placed in a 96-well PCR plate cut at the bottom and put into a container for hydroponic culture in greenhouse. At two-leaf stage, the seedlings were transferred to the International Rice Research Institute (IRRI) nutrient solution with pH 5.5 (Xia et al., 2015). The solution was changed every three days till four-leaf stage, and then the 0.8% NaCl (136.8 mmol/L) was used for salt treatment. In the period of three days, to keep the concentration of solution, we added distilled water to a fixed position that was marked in a container. The nutrient solution without NaCl was used as the control.

The same rice germplasm as the seedling stage was soaked and seeded in a 32-well seedling tray. At four-leaf stage, the seedlings were transplanted into 15 L plastic buckets with soil in a greenhouse. Each plastic bucket contained 5 plants, and at jointing, stage 4 plants with the same growth status were retained. Then, at booting stage, representing the rice entering into reproductive stage, each barrel was treated with 10 L of 0.5% NaCl (85.5 mmol/L). Distilled water was added every day to keep the whole volume. In rice, booting stage could be approximately judged as the pulvinus of flag leaf and penultimate leaf were overlapped (Hoshikawa, 1989; Xu et al., 2020). And so, the date that the pulvinus of flag leaf and penultimate leaf reached together was used to start salt treatment for each accession. Each variety treated by clean water was used as the control.

### Measurement of Salt Tolerance Related Traits or Indices at Seedling and Reproductive Stages

At the seedling stage, on the 2nd, 4th, and 6th days after salt treatment, the second leaf from the top of the seedling was selected and the chlorophyll content of the leaf was measured by SPAD 502 Plus Chlorophyll Meter (Konica Minolta Investment Ltd, Japan). At the reproductive stage, the chlorophyll content of rice flag leaf was determined on the 0, 7th, 14th, and 21st days of salt treatment. On the 6th day after salt treatment, at the seedling stage, the plant height, root length, and fresh weight were measured. On the 4th and 6th days after salt treatment, the number of dead seedlings under salt stress was counted, and then the death rate was calculated. Five to 8 seedlings with the same growth status were selected as a replicate, each treatment along with the control was repeated 3 times.

At the reproductive stage, the flag leaves of rice were selected on the 7th, 14th, and 21st day after salt treatment to determine the chlorophyll fluorescence parameters by FMS 2 + field-portable pulse-modulated chlorophyll fluorometer (Hansatech Instruments, England), respectively. After dark adaptation to 30 min, the initial fluorescence (Fo) was measured with weak measuring light, and then the maximum fluorescence (Fm) was measured with a strong flash (5,000 μmol m^–2^ s^–1^, pulse time 0.7 s). The variable fluorescence (Fv) was calculated as Fv = Fm-Fo, along with the maximum photochemical efficiency of photosystem II (PSII) (Fv/Fm), and the potential photochemical efficiency of PS II (Fv/Fo). After 14 days of salt treatment, the third leaf from the top of each plant was sampled and dried. The dried sample was crushed by a grinder, and 0.1 g of sample was placed in a test tube with a lid, then 10 ml of 100 mM acetic acid was added and bathed at 90°C for 2 h. After cooling and 5,000 rpm centrifugation for 15 min, the supernatant was transferred to the 10 ml centrifuge tube. After proper dilution, the contents of Na and K were determined by atomic absorption spectrophotometer (Pin AAcle 900F, Perkin Elemer Co., Ltd). Panicle length and the number of grains per panicle were measured at the end of grain filling, and the seed setting rate was calculated. Four plants per bucket were set as a replicate. Each treatment along with the control was repeated 3 times.

According to the method of Qi et al. (2005), the ST grade of rice at seedling stage and reproductive development stages were individually classified. The relative salt damage rate (RSD) was calculated as follows: RSD (%) = (control - salt treatment)/control × 100. Additionally, the relative value (RV) of ST was calculated as follows: RV (%) = salt stress value/control value × 100. Accordingly, six ST related indices at the seedling stage and ten ST-related indices at the reproductive stage were abbreviated as Table 2.

**TABLE 2:** Details of salt tolerance (ST) related indices at the seedling stage and reproductive stage.

Indices	Description	Stage
sRV-PH	RV of plant height	Seedling stage
sRV-FW	RV of fresh weight	
sRV-DR4	RV of death rate on the 4^th^ day	
sRV-DR6	RV of death rate on the 6^th^ day	
sRV-Chl4	RV of chlorophyll content on the 4^th^ day	
sRV-Chl6	RV of chlorophyll content on the 6^th^ day	
rRV-Na	RV of Na^+^ content	Reproductive stage
rRV-Na/K	RV of Na^+^/K^+^ content	
rRSD-SR	RSD of seed-setting rate	
rRSD-PL	RSD of panicle length	
rRV-Chl7	RV of chlorophyll content on the 7^th^ day	
rRV-Chl14	RV of chlorophyll content on the 14^th^ day	
rRV-Chl21	RV of chlorophyll content on the 21^st^ day	
rRV-Fv/Fm7	RV of chlorophyll fluorescence parameters (Fv/Fm) on the 7^th^ day	
rRV-Fv/Fm14	RV of chlorophyll fluorescence parameters (Fv/Fm) on the 14^th^ day	
rRV-Fv/Fm21	RV of chlorophyll fluorescence parameters (Fv/Fm) on the 21^st^ day	

Excel 2016 and R 3.6.3 were used for data processing and correlation analysis.

### Genome-Wide Association Study for Rice Salt Tolerance Indices

The publicly available 700K SNP dataset of RDP1 varieties were downloaded^1^ for subsequently GWAS analysis (McCouch et al., 2016). SNP filtering was carried out according to the following steps: (1) SNPs with minor allele frequency (MAF) ≥ 2% and missing rate < 25% were selected; (2) based on gene functional annotations of the “Nipponbare” genome IRGSP-1.0.46.chr.gff3 from the Rice Genome Annotation Project^2^ and The Rice Annotation Project,^3^ SNPs within annotated genes from 2kb upstream to 2kb downstream were extracted through SnpEff (Cingolani et al., 2012). GWAS was performed with the software “GAPIT” and two kinds of statistical models, CMLM and BLINK, were used (Zhang et al., 2010; Huang et al., 2019). A minimum Bayes factor (mBF) was applied to identify significant markers based on the P value threshold for significance. The P (mBF) was calculated using the following formula: mBF = –e*P*ln(P) (Goodman, 2001). Thus, the significance threshold in this study was –log10(P) = 3.78. Manhattan and Q-Q plots were generated with the “CMplot” package in R environment (Yin et al., 2020).

### Principal Component Analysis

Principal component analysis (PCA) was performed using the ST-related indices with the R package ‘‘FactoMineR’’ and visualized with ‘‘factoextra’’.^4,5^

### Differential Gene Expression Analysis

A total of 25 rice salt-tolerant transcriptome datasets from 7 independent studies were collected from the Gene Expression Omnibus (GEO) database.^6^ R package “limma” was used to mine differential expression genes (Ritchie et al., 2015). Transcripts or genes with P-values < 0.05 and absolute value of |log2(fold change)| > 1 were assigned as DEGs.

### Meta-Analysis of Rice Salt Tolerance Quantitative Trait Loci

Information of ST QTLs at seedling and reproductive stages were collected from 18 references published from 2004 to 2020, which included QTL confidence interval, linkage molecular markers, LOD value, phenotypic variation rate (PVE%), and mapping parents (Supplementary Table 24). According to the positions of the molecular markers linked with these ST-responsive QTLs on rice reference genome of Nipponbare, an integrated physical map for all ST responsive QTLs and their linked molecular markers was obtained. Then, based on the ratio of 1 cM per 244 kb (Chen et al., 2002), this integrated physical map was further converted to a virtual genetic map, namely, a consistent genetic map, which included 616 and 134 markers linked with ST QTLs at seedling and reproductive stages, respectively. This conversion is based on the assumption that the ratio of genetic to physical distance is invariant throughout the genome except that in the centromeric regions, which has been used in previous studies for meta-analysis of QTLs related to several traits (Courtois et al., 2009). According to the information of all ST responsive QTLs integrated in this consistent genetic map, the Meta-QTL analysis was performed by using the software BioMercator V4.2 (Veyrieras et al., 2007; Sosnowski et al., 2012). The method relies on a clustering algorithm based on a Gaussian mixture model and enables the determination of the probable number of clusters considered as the “true” QTLs underlying the QTLs observed in a given region. The optimal number of clusters was chosen by means of an information-based criterion, and the position and confidence interval of the meta quantitative ST loci (MqST) was then estimated.

### Gene Transcription Analysis Under Salt Stress

The qRT-PCR experiments were performed to examine the transcription levels of 7 genes in 15 accessions after treatment (HAT) with salt. Both favorable and unfavorable alleles in each of these 7 genes were included in the 15 accessions (Supplementary Table 27). The leaves of four-leaf stage seedlings of each accession were harvested at 12 and 24 h after 0.8% NaCl treatment, respectively, and the leaves without NaCl treatments was used as the control. All samples were immediately snap-frozen in liquid nitrogen.

Each RNA sample was isolated using the Mini BEST Plant RNA Extraction Kit (TaKaRa, Dalian, China) in accordance with the protocol of the manufacturer, and then treated with gDNA Eraser (TaKaRa, Dalian, China) following the instructions of the manufacturer to eliminate any contaminant gDNA. The treated RNA solution (10 μl) was subjected to reverse transcriptase reactions with PrimeScript™ Reverse Transcriptase Reagent Kit with gDNA Eraser (Perfect Real Time) (TaKaRa, Dalian, China) in accordance with the protocol of the manufacturer. Gene-specific primers were designed using Primer 5.0 (Supplementary Table 27). *Actin* was used as the internal reference gene. Quantitative RT-PCR was performed using a Bio-Rad CFX96™ Real-Time System (Bio-Rad, United States) using the SYBR Premix Ex Taq™ Kit (Perfect Real Time) (TaKaRa, Japan) in accordance with the protocol of the manufacturer. qRT-PCR conditions were as follows: 30 s at 94°C for denaturation, 40 cycles for 5 s at 94 °C, 30 s at 56 °C, and 10 s at 72 °C. The expression levels of target genes were calculated with the 2^–ΔΔ*Ct*^ comparative threshold cycle (Ct) method. All reactions were performed in three biological replicates, and the results of Ct values were determined with Bio-Rad CFX Manager V1.6.541.1028 software. Relative expression level of each target genes was calculated as follows: expression levels of salt stress/expression levels of control.

## Data Availability Statement

The original contributions presented in the study are included in the article/Supplementary Material, further inquiries can be directed to the corresponding author.

## Author Contributions

SZu designed and supervised the works and critically commented and revised it. GC, KH, and JZo performed most of the experiments, analyzed the experimental data, and wrote the manuscript. FG, WS, QJ, JZn, ZG, ZF, ZC, XW, and SZh conducted a part of experiments and analyzed part of the data. All authors read and approved the final manuscript.

## Conflict of Interest

The authors declare that the research was conducted in the absence of any commercial or financial relationships that could be construed as a potential conflict of interest.

## Publisher’s Note

All claims expressed in this article are solely those of the authors and do not necessarily represent those of their affiliated organizations, or those of the publisher, the editors and the reviewers. Any product that may be evaluated in this article, or claim that may be made by its manufacturer, is not guaranteed or endorsed by the publisher.

## Footnotes

1
http://www.ricediversity.org/data/

2
http://rice.plantbiology.msu.edu/

3
https://rapdb.dna.affrc.go.jp

4
https://CRAN.R-project.org/package=FactoMineR

5
https://CRAN.R-project.org/package=factoextra

6
https://www.ncbi.nlm.nih.gov/geo/

## Supplementary Material

Supplementary Figure 1 Trait distribution of the accessions on each salt tolerance (ST)-related index at seedling stage. The boxes with 3 lines represent first quartile, median and third quartile. Plotlines extending from the boxes (whiskers) indicate variability outside the upper and lower quartiles. Each point represents a variety, and the black points represent outliers.

Supplementary Figure 2 Trait distribution of the accessions on each ST related index at reproductive stage. The boxes with 3 lines represent first quartile, median and third quartile. Plotlines extending from the boxes (whiskers) indicate variability outside the upper and lower quartiles. Each point represents a variety, and the black points represent outliers.

Supplementary Figure 3 Comparison of mean value of each ST index among 5 subgroups at seedling and reproductive stages. Error bars mean the standard errors. Different lowercase letters represent significant difference at the P value of 0.05, and “NS” means no significant difference (Kruskal-Wallis one-way ANOVA, Bonferroni correction). Aromatic subgroup was not included in this analysis because the samples are too less.

Supplementary Figure 4 Distribution of gene-based/tightly linked SNP markers in rice genome. Different colors represent the density of SNP markers within 1Mb window size on the chromosome.

Supplementary Figure 5 Correlation of the accessions accumulated the number of low frequency favorable haplotypes and the ST indices value at the seedling stage. The dotted line and gray region represent the linear regression and 95% CI, respectively.

Supplementary Figure 6 Correlation of the accessions accumulated the number of low frequency favorable haplotypes and the ST indices value at the reproductive stage. The dotted line and gray region represent the linear regression and 95% CI, respectively.

Supplementary Figure 7 Transcription levels of 7 genes in 15 accessions at 12 and 24 hours after salt treatment. Error bars mean the standard errors. ** indicate the difference reached statistically significant difference at the P value < 0.01.
