# Supplementary material for: Genome-Wide Association Analysis for Salt–Induced Phenotypic and Physiologic Responses in Rice at Seedling and Reproductive Stages
Source: Front Plant Sci. 2022 Feb 9;13:822618. doi: 10.3389/fpls.2022.822618 (PMC8863738; doi:10.3389/fpls.2022.822618)
Supplement: Supplementary Figure 1 — Trait distribution of the accessions on each salt tolerance (ST)-related index at seedling stage. The boxes with 3 lines represent first quartile, median and third quartile. Plotlines extending from the boxes (whiskers) indicate variability outside the upper and lower quartiles. Each point represents a variety, and the black points represent outliers. [file Presentation_1.PPTX]

## Slide 1
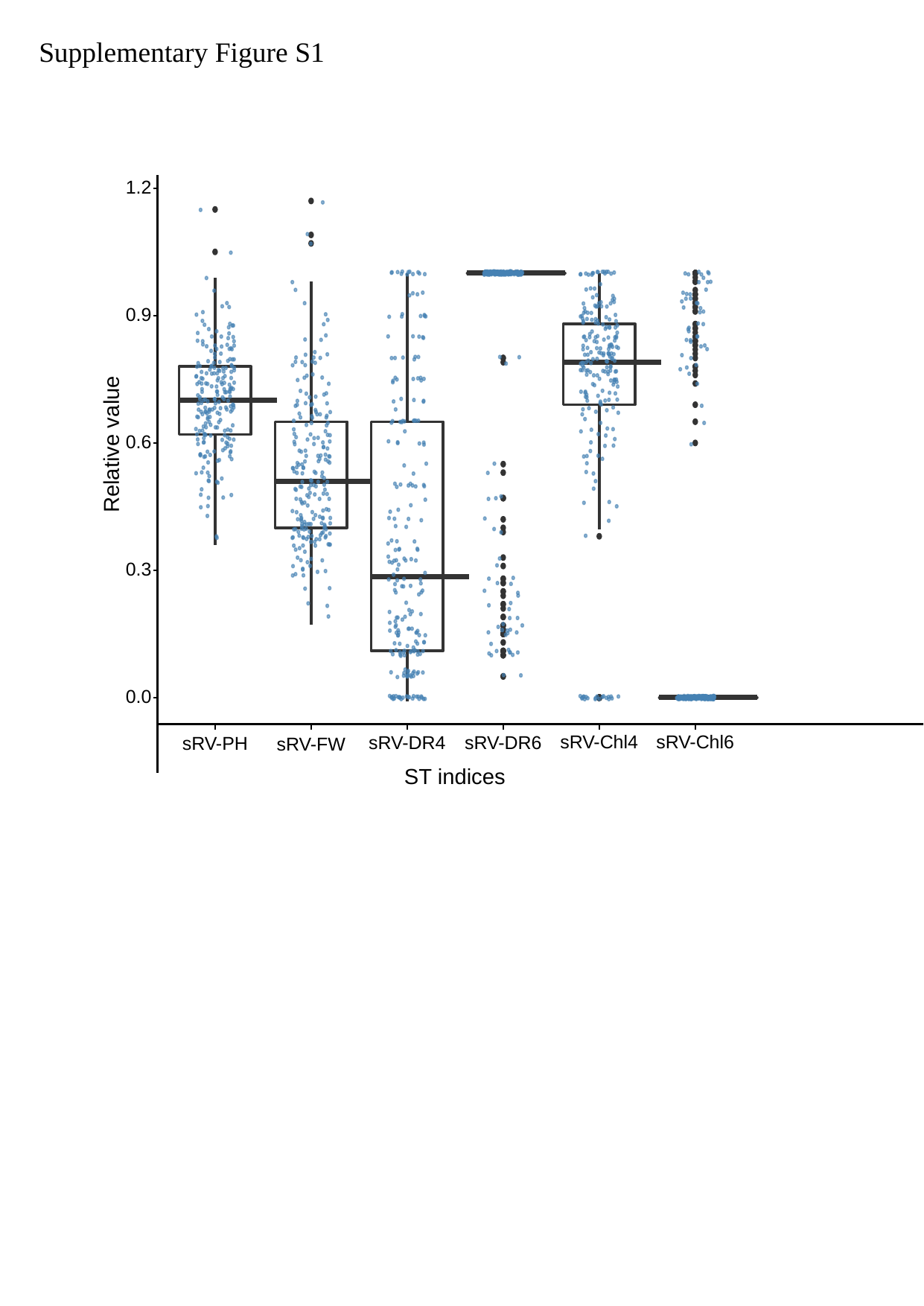

Supplementary Figure S1
1.2
0.9
0.6
Relative value
0.3
0.0
sRV-Chl6
sRV-Chl4
sRV-DR6
sRV-DR4
sRV-PH
sRV-FW
ST indices

## Slide 2
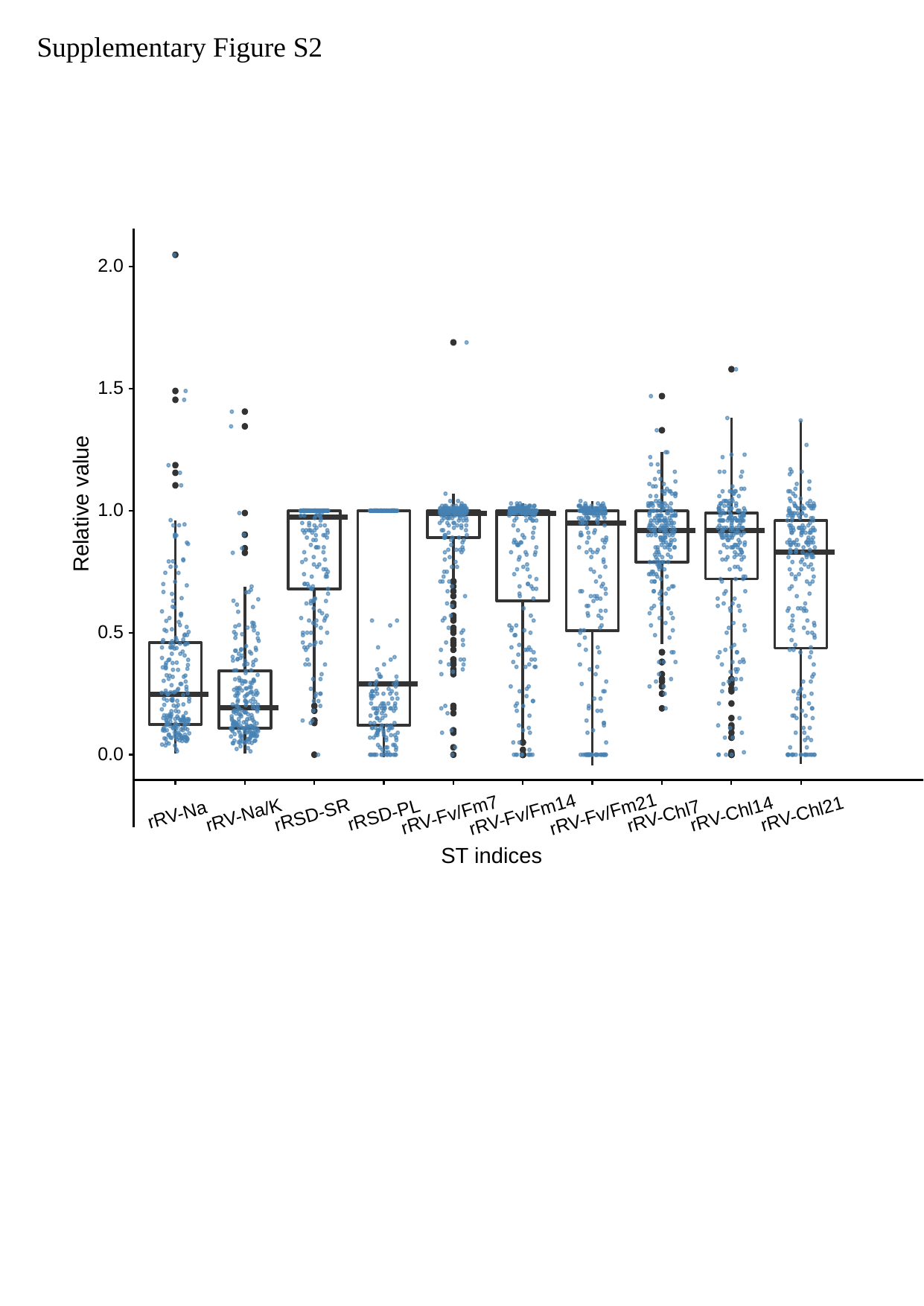

Supplementary Figure S2
2.0
1.5
Relative value
1.0
0.5
0.0
rRV-Fv/Fm21
rRV-Chl14
rRV-Chl21
rRV-Fv/Fm14
rRV-Fv/Fm7
rRV-Chl7
rRV-Na
rRSD-PL
rRSD-SR
rRV-Na/K
ST indices

## Slide 3
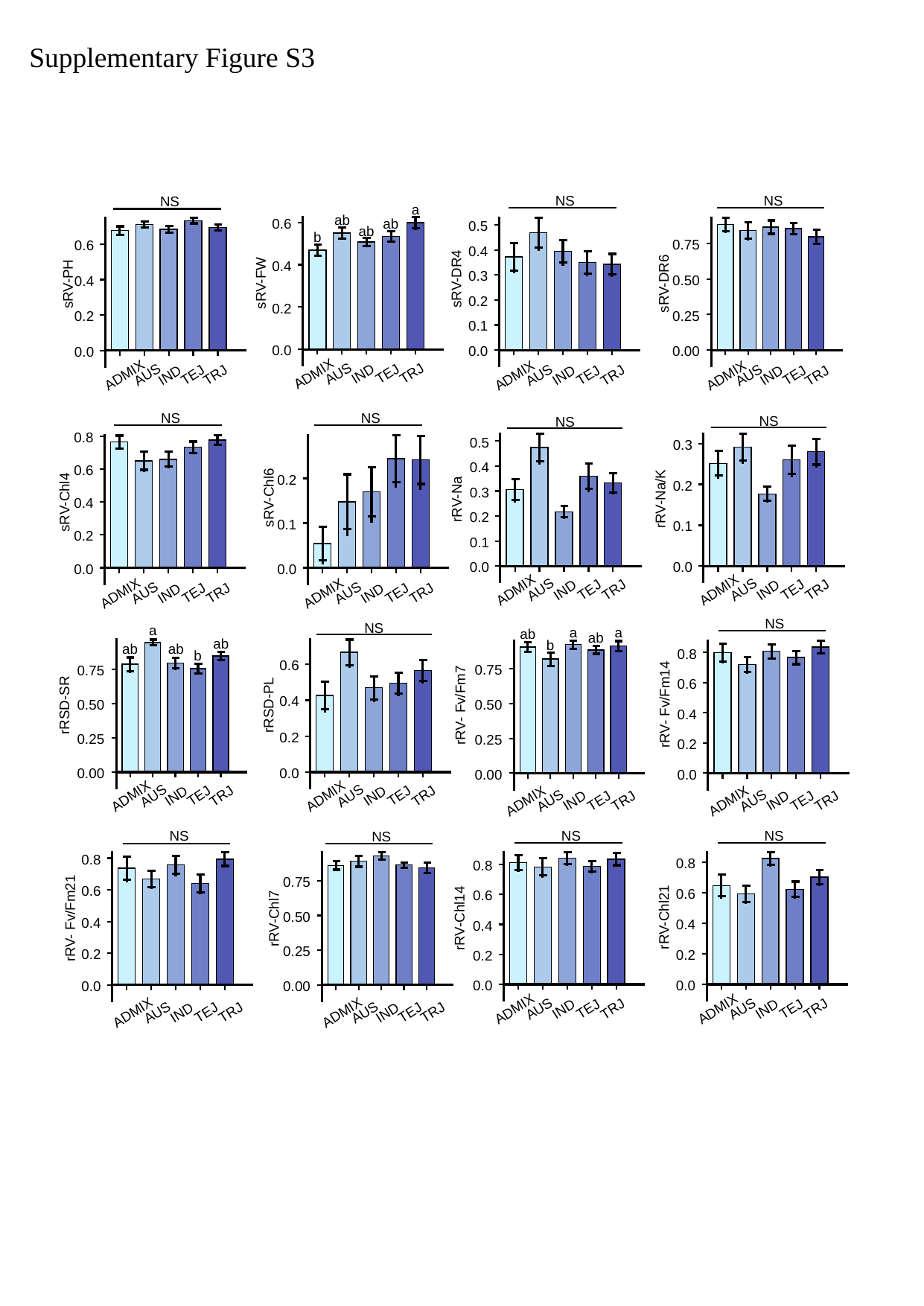

Supplementary Figure S3
NS
0.5
0.4
0.3
sRV-DR4
0.2
0.1
0.0
AUS
TEJ
TRJ
ADMIX
IND
NS
0.75
0.50
sRV-DR6
0.25
0.00
AUS
TEJ
TRJ
ADMIX
IND
NS
0.6
0.4
sRV-PH
0.2
0.0
AUS
TEJ
TRJ
ADMIX
IND
a
ab
0.6
ab
ab
b
0.4
sRV-FW
0.2
0.0
AUS
TEJ
TRJ
ADMIX
IND
NS
0.8
0.6
sRV-Chl4
0.4
0.2
0.0
AUS
TEJ
TRJ
ADMIX
IND
NS
0.2
sRV-Chl6
0.1
0.0
AUS
TEJ
TRJ
ADMIX
IND
NS
0.3
0.2
rRV-Na/K
0.1
0.0
AUS
TEJ
TRJ
ADMIX
IND
NS
0.5
0.4
0.3
rRV-Na
0.2
0.1
0.0
AUS
TEJ
TRJ
IND
ADMIX
NS
0.8
0.6
rRV- Fv/Fm14
0.4
0.2
0.0
AUS
TEJ
TRJ
IND
ADMIX
NS
0.6
0.4
rRSD-PL
0.2
0.0
AUS
TEJ
TRJ
ADMIX
IND
a
ab
ab
ab
b
0.75
rRSD-SR
0.50
0.25
0.00
AUS
TEJ
TRJ
ADMIX
IND
0.75
rRV- Fv/Fm7
0.50
0.25
0.00
AUS
TEJ
TRJ
ADMIX
IND
NS
0.8
0.6
rRV-Chl21
0.4
0.2
0.0
AUS
TEJ
TRJ
IND
NS
0.8
0.6
rRV-Chl14
0.4
0.2
0.0
AUS
TEJ
TRJ
ADMIX
IND
NS
0.8
0.6
rRV- Fv/Fm21
0.4
0.2
0.0
AUS
TEJ
TRJ
ADMIX
IND
NS
0.75
0.50
rRV-Chl7
0.25
0.00
AUS
TEJ
TRJ
IND
ADMIX
ADMIX
a
a
ab
ab
b

## Slide 4
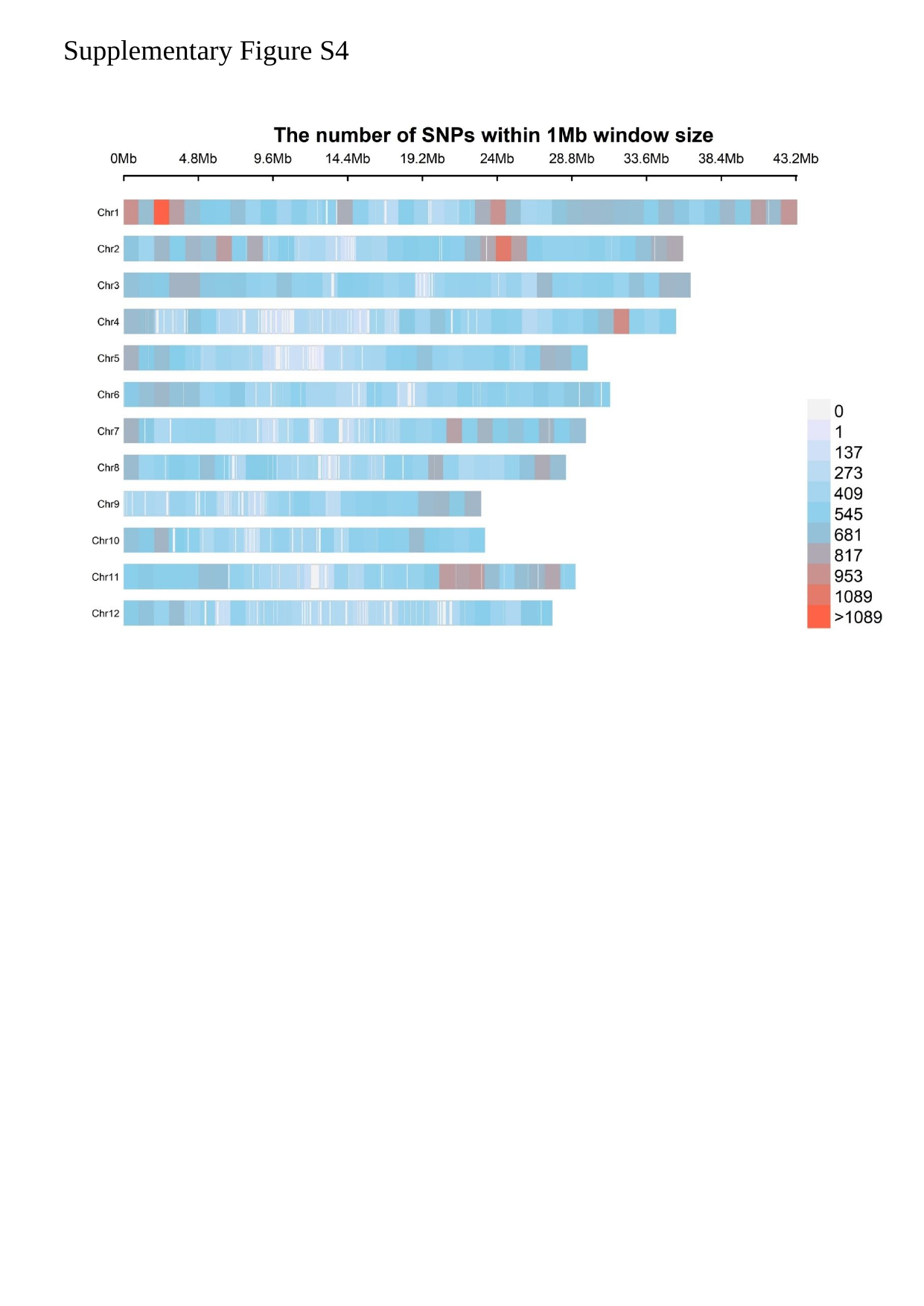

Supplementary Figure S4

## Slide 5
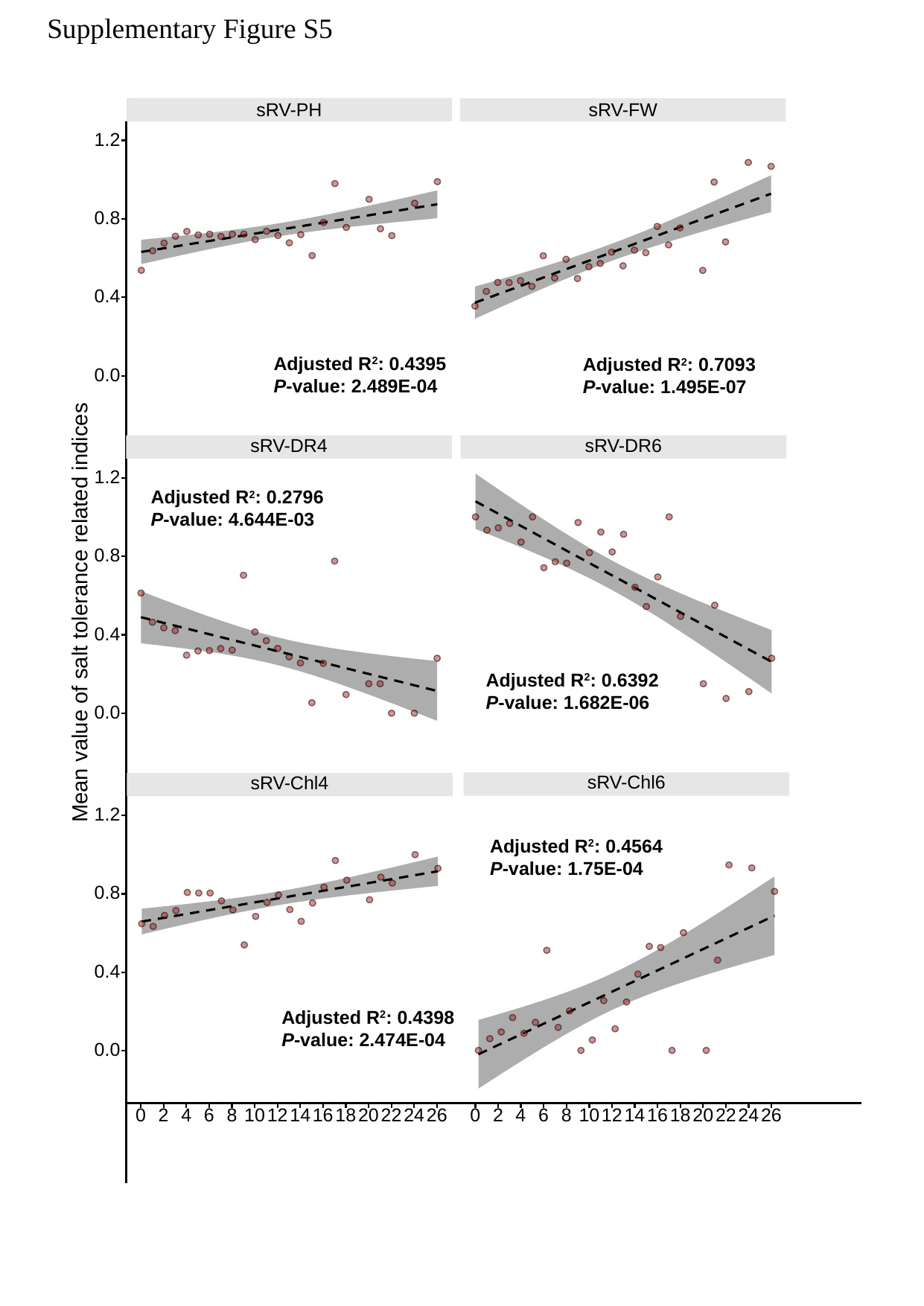

Supplementary Figure S5
sRV-PH
sRV-FW
1.2
0.8
0.4
Adjusted R2: 0.4395
P-value: 2.489E-04
Adjusted R2: 0.7093
P-value: 1.495E-07
0.0
sRV-DR6
sRV-DR4
1.2
Adjusted R2: 0.2796
P-value: 4.644E-03
0.8
Mean value of salt tolerance related indices
0.4
Adjusted R2: 0.6392
P-value: 1.682E-06
0.0
sRV-Chl6
sRV-Chl4
1.2
Adjusted R2: 0.4564
P-value: 1.75E-04
0.8
0.4
Adjusted R2: 0.4398
P-value: 2.474E-04
0.0
0
6
8
10
16
18
20
26
0
6
8
10
16
18
20
26
2
12
14
22
24
2
12
14
22
24
4
4

## Slide 6
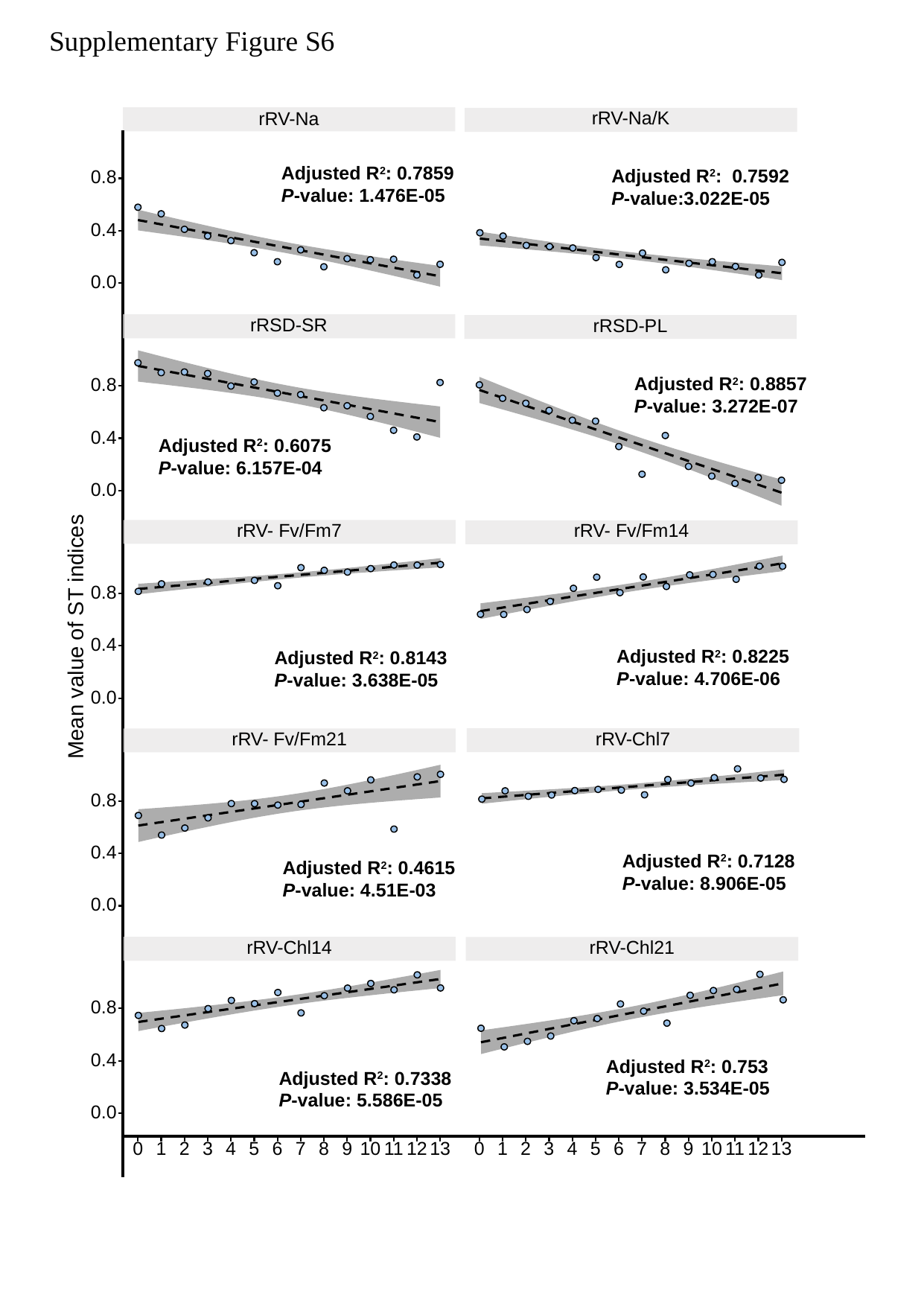

Supplementary Figure S6
rRV-Na/K
rRV-Na
Adjusted R2: 0.7859
P-value: 1.476E-05
Adjusted R2: 0.7592
P-value:3.022E-05
0.8
0.4
0.0
rRSD-SR
rRSD-PL
Adjusted R2: 0.8857
P-value: 3.272E-07
0.8
Adjusted R2: 0.6075
P-value: 6.157E-04
0.4
0.0
rRV- Fv/Fm7
rRV- Fv/Fm14
0.8
Mean value of ST indices
Adjusted R2: 0.8225
P-value: 4.706E-06
0.4
Adjusted R2: 0.8143
P-value: 3.638E-05
0.0
rRV-Chl7
rRV- Fv/Fm21
0.8
Adjusted R2: 0.7128
P-value: 8.906E-05
0.4
Adjusted R2: 0.4615
P-value: 4.51E-03
0.0
rRV-Chl14
rRV-Chl21
0.8
Adjusted R2: 0.753
P-value: 3.534E-05
0.4
Adjusted R2: 0.7338
P-value: 5.586E-05
0.0
3
13
3
13
0
6
8
9
10
0
6
8
9
10
1
2
11
12
1
2
11
12
5
5
4
4
7
7

## Slide 7
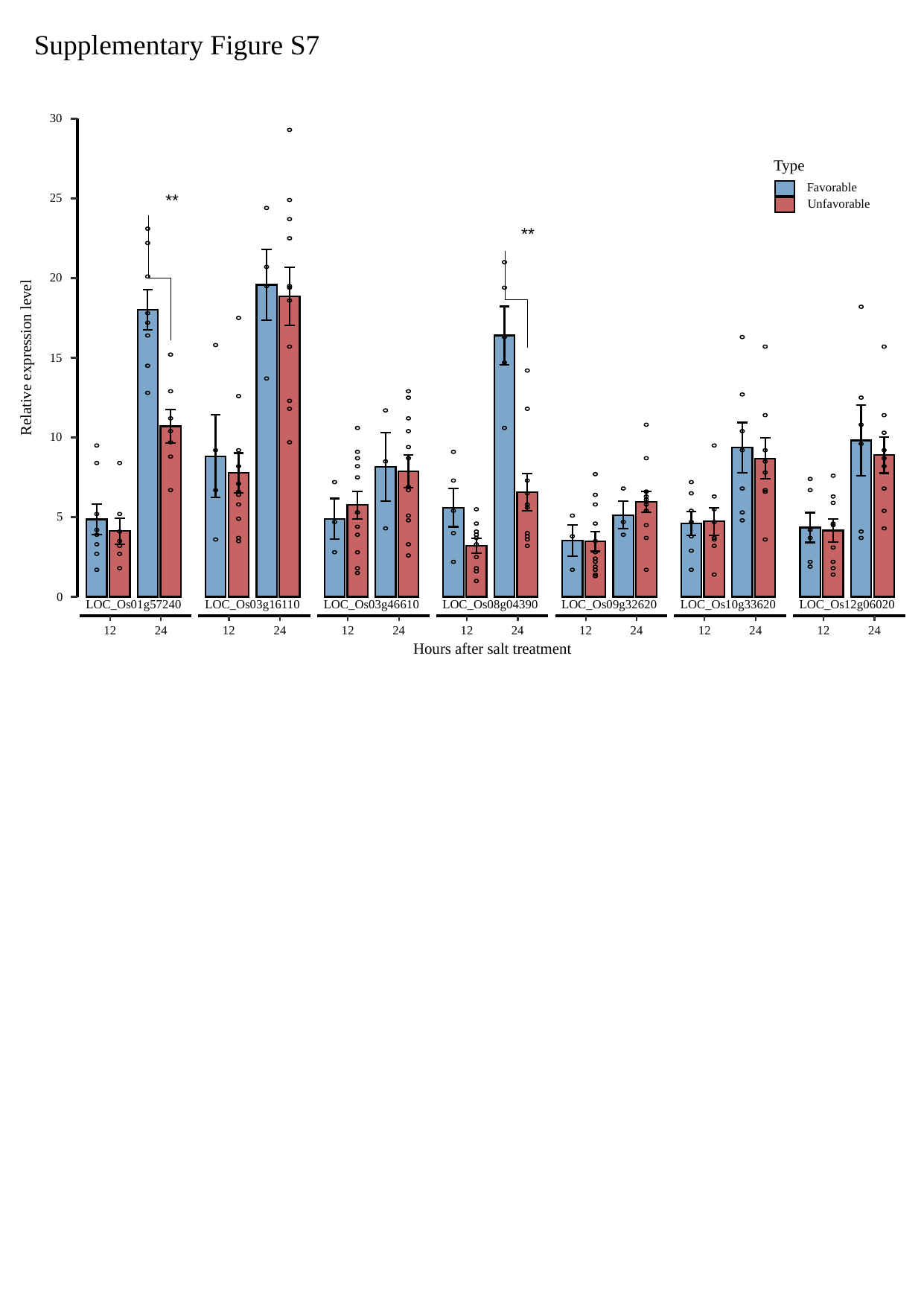

Supplementary Figure S7
30
Type
Favorable
Unfavorable
**
25
**
20
Relative expression level
15
10
5
0
LOC_Os01g57240
LOC_Os03g16110
LOC_Os03g46610
LOC_Os08g04390
LOC_Os09g32620
LOC_Os10g33620
LOC_Os12g06020
12
24
12
24
12
24
12
24
12
24
12
24
12
24
Hours after salt treatment
